# Pharmacovigilance in vaccines

**DOI:** 10.4103/0253-7613.64488

**Published:** 2010-04

**Authors:** Salil Budhiraja, Raghuram Akinapelli

**Affiliations:** Department of Pharmacovigilance and Drug Safety, MakroCare, Hyderabad – 500 033, Andhra Pradesh, India

Sir,

Vaccines are among the safest tools of modern medicine that help in protecting against disease by inducing immunity. In all countries, routine immunization represents a fundamental service of the public health system, offering one of the most cost-effective methods of reducing morbidity and mortality among children less than five. A lack of complete vaccine coverage increases the risk of disease for the entire population. Pharmacovigilance is the science related to the safety of drugs and it helps prevent harmful effects of the drugs in population. It originated with thalidomide tragedy that caused 10000 birth defects in children. Regulatory systems were established soon to bring an improvement in post-marketing surveillance for the safer medicines.[1]

Since the majority of vaccines are administered to a vulnerable (children) as well as healthy population, a strict safety supervision of vaccines is essential. Safety issues in vaccines may lead to rumors and undermine a general confidence in vaccination and, ultimately, have dramatic consequences on immunization coverage and disease incidence.[2] Safety of vaccines, therefore, must be excellent to make it acceptable, since it usually has a deferred individual benefit, but immediate adverse drug reactions (ADRs). Vaccine pharmacovigilance is defined as the science and activities relating to the detection, assessment, understanding, prevention, and communication of adverse events following immunization, or of any other vaccine- or immunization-related issues. The WHO defines an adverse event following immunization (AEFI) as "a medical incident that takes place after an immunization causes concern, and is believed to be caused by the immunization." The goal of vaccine pharmacovigilance is the early detection and timely response to adverse events following immunization, in order to minimize negative effects to the health of individuals and lessen the potential negative impact on immunization of population.

Although vaccines are considered to be medicines with anti-infective activity that work by immunological action and administered for prophylaxis, pharmacovigilance for vaccines should be different from other drugs because of the following reasons.

1. *Information to be captured is complex and different from other drugs*.
  Vaccines are complex biological products, which may include multiple antigens, live organisms, adjuvants, and preservatives. ADRs may be due to administration of live wild viruses e.g. lymphocyte meningitis after anti-mumps vaccine or may be non-specific, related to a component different from the antigen (aluminum hydroxide involved in the "macrophagic myofasciitis," allergic reactions to neomycin, latex, egg, or gelatin).[3] So, each component has unique safety implications, which require different information to capture as compared to other drugs. In a vaccination campaign, the vaccine is administered to a large population over a short period of time, which may result in administration error and hence has to be dealt with differently.
2. Different modes of causality assessment
  There are some issues, which make evaluation of vaccines different from other drugs. Local or immediate adverse reactions, which occur due to administrative error and attenuated virus, can be attributed with a degree of confidence but delayed events are difficult to correlate. They have immunological considerations in addition to pharmacological action and take a long time to respond.[4] Criteria commonly used to determine causality such as resolution of the event following treatment discontinuation and the result of re-challenge cannot be used to assess causality of an event occurring after vaccination.[5]
3. Reporters and reporting chain
  Vaccines are a part of national health plan and physicians involved in primary health care conduct the immunization en masse. So, reporter and reporting chain are different, especially in developing countries where physicians involved in primary health care are not exposed to technology (internet, etc) and pharmacovigilance sensitization.
  Although pharmacovigilance of vaccines is important, yet it is given much less attention. There are no different systems and regulatory guidelines in most of the countries for this activity. Lot of research is going on for vaccine development in infectious diseases e.g. HIV, malaria, H1N1, etc that have a high patient load. For the last two decades, pharmacovigilance has been gaining an increasing attention. It is now, high time that vaccines also receive their due attention.
